# Variation in care and outcomes for people after hip fracture with and without cognitive impairment; results from the Australian and New Zealand Hip Fracture Registry

**DOI:** 10.1016/j.jnha.2023.100030

**Published:** 2024-01-04

**Authors:** Morag E. Taylor, Lara A. Harvey, Maria Crotty, Ian A. Harris, Catherine Sherrington, Jacqueline C.T. Close

**Affiliations:** a Falls, Balance and Injury Research Centre, Neuroscience Research Australia, Sydney, New South Wales, Australia; b Population Health, Faculty of Medicine and Health, University of New South Wales, Sydney, New South Wales, Australia; c UNSW Ageing Futures Research Institute, Sydney, New South Wales, Australia; d College of Medicine and Public Health, Flinders University, Adelaide, South Australia, Australia; e Rehabilitation Unit, Southern Adelaide Local Health Network, Adelaide, South Australia, Australia; f Ingham Institute for Applied Medical Research, South Western Sydney Clinical School, UNSW Sydney, Liverpool Hospital, Liverpool, NSW, Australia; g Sydney Musculoskeletal Health, Institute for Musculoskeletal Health, The University of Sydney and Sydney Local Health District, Sydney, New South Wales, Australia; h Sydney School of Public Health, Faculty of Medicine and Health, The University of Sydney, Sydney, New South Wales, Australia; i School of Clinical Medicine, Faculty of Medicine and Health, University of New South Wales, Sydney, New South Wales, Australia

**Keywords:** Hip fracture, Dementia, Cognitive dysfunction, Mortality, Benchmarking, Clinical guidelines

## Abstract

**Background:**

People with dementia have poorer outcomes after hip fracture and this may be due in part to variation in care. We aimed to compare care and outcomes for people with and without cognitive impairment after hip fracture.

**Methods:**

Retrospective cohort study using Australian and New Zealand Hip Fracture Registry data for people ≥50 years of age who underwent hip fracture surgery (n = 49,063). Cognitive impairment or known dementia and cognitively healthy groups were defined using preadmission cognitive status. Descriptive statistics and multivariable mixed effects models were used to compare groups.

**Results:**

In general, cognitively impaired people had worse care and outcomes compared to cognitively healthy older people. A lower proportion of the cognitively impaired group had timely pain assessment (≤30 min of presentation: 61% vs 68%; *p* < 0.0001), were given the opportunity to mobilise (89% vs 93%; *p* < 0.0001) and achieved day-1 mobility (34% vs 58%; *p* < 0.0001) than the cognitively healthy group. A higher proportion of the cognitively impaired group had delayed pain management (>30 mins of presentation: 26% vs 20%; p < 0.0001), were malnourished (27% vs 15%; *p* < 0.0001), had delirium (44% vs 13%; *p* < 0.0001) and developed a new pressure injury (4% vs 3%; *p* < 0.0001) than the cognitively healthy group. Fewer of the cognitively impaired group received rehabilitation (35% vs 64%; *p* < 0.0001), particularly patients from RACFs (16% vs 39%; *p* < 0.0001) and were prescribed bone protection medication on discharge (24% vs 27%; p < 0.0001). Significantly more of the cognitively impaired group had a new transfer to residential care (46% vs 11% from private residence; *p* < 0.0001) and died at 30-days (7% vs 3% from private residence; 15% vs 10% from RACF; both *p* < 0.0001). In multivariable models adjusting for covariates with facility as the random effect, the cognitively impaired group had a greater odds of being malnourished, not achieving day-1 walking, having delirium in the week after surgery, dying within 30 days, and in those from private residences, having a new transfer to a residential care facility than the cognitively healthy group.

**Conclusions:**

We have identified several aspects of care that could be improved for patients with cognitive impairment – management of pain, mobility, nutrition and bone health, as well as delirium assessment, prevention and management strategies and access to rehabilitation. Further research is needed to determine whether improvements in care will reduce hospital complications and improve outcomes for people with dementia after hip fracture.

## 1 Introduction

Approximately 30% of all patients admitted to hospital with a hip fracture have an additional diagnosis of cognitive impairment/dementia [1]. Hospital acquired complications, particularly delirium, are more common in people with dementia who fracture their hip (i.e. 1.5–3.6 times the odds of infection, hip joint dislocation, respiratory complications and delirium than people without dementia) [2]. After hip fracture, people with dementia are less likely to regain their walking ability and twice as likely to move into residential care and die compared to people without dementia [[2], [3], [4]]. Previous research has demonstrated that hospital acquired complications increase length of stay and healthcare costs and, reduce functional recovery and quality of life [5,6]. Hospital acquired complications also have funding penalties in Australia i.e. hospitals receive less government money when patients experience complications [7].

The significant number of hospital acquired complications (e.g. 58% with delirium [8]) in people with dementia may be due to variation in care. For example, in the UK, people with hip fracture and comorbid dementia were less likely to be mobilised early after surgery compared to patients without dementia (72% vs 82%) [9]. Early mobilisation for patients with dementia was associated with 83% increased odds of being discharged at 30-days [9]. In 2014, the Australian and New Zealand Guideline for Hip Fracture Care: Improving Outcomes in Hip Fracture Management of Adults was published. This informed the development of the Hip Fracture Care Clinical Care Standard (2016) with the aim of supporting the delivery of high-quality care to patients presenting to hospital with a hip fracture [10]. The Hip Fracture Care Clinical Care Standard presents seven quality statements, with one or more quality indicators for each statement. The Australian and New Zealand Hip Fracture Registry (ANZHFR) is a clinical quality registry that collects data about the care and outcomes for people admitted to hospital with a hip fracture and benchmarks performance against the Hip Fracture Care Clinical Care Standard [10,11]. In 2020, 86 hospitals entered patient-level data which includes 82% of eligible public hospitals in Australia and New Zealand. The ANZHFR releases annual reports that are publicly available, but little information is available about specific patient populations e.g. people with dementia [12].

A recent Cochrane review concluded that there was a lack of robust evidence to support models of care including enhanced rehabilitation for people with dementia hospitalised with a hip fracture [13]. This review also concluded that research should aim to determine the approaches needed to improve outcomes [13]. People with dementia have unique care needs due to the high incidence of delirium/hospital acquired complications, injury- and surgery-related mobility limitations and reduced cognitive and physical reserves. This, together with the demonstrated lack of evidence for effective interventions, suggests that an intervention needs to be developed and tested specifically for people with dementia who sustain a hip fracture. This intervention should be informed by a better understanding of current care and outcomes for people with cognitive impairment or dementia. We therefore aimed to compare care provision and outcomes in patients with and without cognitive impairment undergoing surgery for a hip fracture in Australia and New Zealand.

## 2 Methods

### 2.1 Study design

Retrospective cohort study using ANZHFR data (2016–2020 inclusive).

### 2.2 Participants

ANZHFR collects data on all patients over 50 years of age admitted to a participating hospital with a low trauma hip fracture. Participants aged 50 years and over were classified as cognitively normal or impaired based on the variable ‘pre-admission cognitive status’. Pre-admission cognitive status is coded as normal, impaired cognition or known dementia and unknown. Individuals with missing or unknown pre-admission cognitive status were excluded. Atypical and pathological fractures and patients who did not undergo surgery were also excluded.

### 2.3 Recruitment, consent and ethics

This study used data collected by the ANZHFR. The ANZHFR is a clinical quality registry that has ethical approval from a HREC in each Australian state and New Zealand. This study was approved by the UNSW Human Research Ethics Advisory Panel (HC210311) to use the ANZHFR data for this specific project.

### 2.4 ANZHFR data

The number of hospitals reporting patient-level data has increased over time as more hospitals gain approval to participate. There were 34 hospitals reporting patient-level data in 2016, 56 in 2017, 67 in 2018, 76 in 2019 and 86 in 2020. Data collection includes basic demographics (age, sex, usual place of residence, patient type [e.g. public/private]), preadmission cognitive status, preoperative cognitive assessment (from 2017), preoperative medical assessment, pain assessment and management (from 2017), admission and discharge dates, type of fracture, surgical details (date, operation performed, presence of consultant, type of anaesthesia including nerve block use, surgical delay and reason), weightbearing status, walking ability (pre and post fracture), bone medication (pre and post fracture), pressure injuries, first day mobility/walking offered/achieved (achieved from 2020), new pressure injury during hospital stay (stage 2 or higher), assessed by geriatric medicine, delirium assessment (from 2018), clinical malnutrition assessment (from 2019) and discharge destination. The data dictionary is available on the ANZHFR website: https://anzhfr.org/data-forms/.

The ANZHFR data is linked to the National Death Index (NDI) by the Australian Institute for Health and Welfare (AIHW).

### 2.5 Statistical analysis

Data were analysed with SAS Enterprise Guide version 8.2. Categorical variables are reported as frequency with percent and between cognitive group differences were analysed using Chi squared statistics. Mann-Whitney U and t-tests were used to analyse between cognitive group differences in continuous variables which are reported as mean and standard deviation (SD) or median and interquartile range (IQR), as appropriate. Bone protection medication use (admission and discharge), received rehabilitation, length of stay and mortality are reported for the whole sample by cognitive group, but also in subgroups (by preadmission residential status), as we wanted to explore if care and outcomes differed based on residential status. Multivariable mixed effects logistic regression models were used to examine the association between cognitive impairment/known dementia and 1) being malnourished, 2) not achieving day-1 walking (in those who were mobile preadmission; data only available from 2020), 3) delirium in the week after surgery (in those assessed), 4) new transfer to an RACF (vs home) in people from private residences and 5) 30-day mortality. Missing data were left missing and are reported in Table footnotes.

## 3 Results

**Fig. 1:**
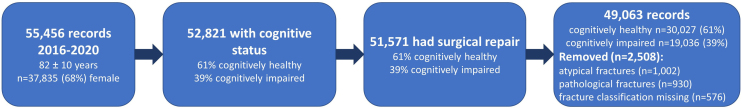
Sample size and cohort selection description.

There were 55,456 records for individuals admitted with a low trauma hip fracture who were ≥50 years of age (Fig. 1). Records without known cognitive status, that did not have surgery and with atypical, pathological or missing fracture classification were removed, leaving 49,063 records reported on hereafter (Fig. 1).

### 3.1 Baseline characteristics

The characteristics of the study cohort are described in Table 1. The cognitively impaired group were older, more likely to live in a residential aged care facility (RACF), be malnourished, had higher comorbidities (ASA grades) and had poorer preadmission walking ability (Table 1). The cognitively healthy group were less likely to be taking any bone protection medication on admission but the pattern of bone protection medication use was similar in patients from private residences and RACF (Table 1).

**Table 1:** Baseline characteristics of people with and without cognitive impairment: ≥50 years of age with surgical repair of a low trauma hip fracture who were admitted to a hospital participating in the Australian and New Zealand Hip Fracture Registry (n = 49,063).

Characteristic, n (%) or mean ± SD	Cognitively healthy	Cognitively impaired	*p*-value
	n = 30,027	n = 19,036	
**Hip fracture registry**			
Australian	23,275 (77.5)	14,655 (77.0)	0.174
New Zealand	6,752 (22.5)	4,381 (23.0)	
**Demographics**			
Age	80.2 ± 10.2	85.9 ± 8.0	<0.0001
Age group			<0.0001
50−64 years	2,505 (8.3)	350 (1.8)	
65−74 years	5,845 (19.5)	1,297 (6.8)	
75−84 years	10,066 (33.5)	5,362 (28.2)	
85−94 years	10,012 (33.3)	9,953 (52.3)	
95+ years	1,599 (5.3)	2,074 (10.9)	
Female	20,642 (68.8)	13,061 (68.7)	0.366
Usual residence			
Private	27,349 (91.6)	7,400 (39.1)	<0.0001
RACF	2,430 (8.1)	11,429 (60.3)	
Other	78 (0.3)	114 (0.6)	
Patient type			
Public	17,077 (76.7)	11,526 (82.3)	<0.0001
Private	4,934 (22.2)	2,451 (17.5)	
Overseas	269 (1.2)	31 (0.2)	
**Preadmission walking ability**			<0.0001
Unaided	17,312 (58.1)	5,163 (27.7)	
Stick or crutch	4,269 (14.3)	1,801 (9.7)	
Two aids or frame	7,863 (26.4)	10,633 (57.0)	
Immobile	365 (1.2)	1,061 (5.7)	
**ASA classification**			<0.0001
1 Healthy	747 (2.8)	85 (0.5)	
2 Mild systemic disease	6,642 (24.8)	959 (5.6)	
3 Severe systemic disease	15,143 (56.5)	10,652 (62.5)	
4 Incapacitating systemic disease	4,193 (15.7)	5,258 (30.9)	
5 Moribund, not expected to survive 24 h	58 (0.2)	89 (0.5)	
**Fracture type**			0.001
Intracapsular	14,747 (49.3)	9,065 (47.7)	
Extracapsular	15,195 (50.7)	9,919 (52.3)	
**Clinical malnutrition assessment**			<0.0001
Not assessed	4,741 (33.5)	2,946 (33.6)	
Malnourished	2,170 (15.3)	2,402 (27.4)	
Not malnourished	7,235 (51.2)	3,411 (38.9)	
**Bone protection medication on admission**			<0.0001
No	20,031 (68.0)	9,908 (53.1)	
Calcium and/or vit D	6,622 (22.5)	7,142 (38.3)	
Yes, prescribed	2,801 (9.5)	1,617 (8.7)	
**Bone protection medication on admission from a private residence**			<0.0001
No	18,702 (69.7)	4,535 (62.7)	
Calcium and/or vit D	5,590 (20.8)	2,053 (28.4)	
Yes, prescription	2,542 (9.5)	650 (9.0)	
**Bone protection medication on admission from an RACF**			0.0003
No	1,171 (49.2)	5,256 (46.8)	
Calcium and/or vit D	968 (40.6)	5,019 (44.7)	
Yes, prescription	243 (10.2)	856 (8.5)	

*Note.* RACF = residential aged care facility.

Missing data: sex n = 29; usual residence n = 263; patient type n = 12,775; preadmission walking n = 596; ASA classification n = 4,902; fracture type n = 137; clinical malnutrition assessment n = 2,111 of 25,016 (variable introduced 2019); bone protection medication on admission n = 942.

### 3.2 Clinical care

A lower proportion of patients in the cognitively impaired group had a pain assessment in <30 min (Table 2). Paramedics were less likely to have given pain relief to patients with known cognitive impairment and a higher proportion received pain relief more than 30 min after presentation when compared to the cognitively healthy group (Table 2). Time to surgery was not different between the groups but more people in the cognitively impaired group underwent general or combined general/spinal anaesthesia. The consultant was present for fewer surgical procedures in the cognitively impaired group, who were also less likely to receive a total hip replacement (Table 2). Weightbearing was unrestricted more often in the group with cognitive impairment than the cognitively healthy group, though high proportions were unrestricted in both groups (Table 2). In patients who were mobile preadmission, a smaller proportion of the group with cognitive impairment were given the opportunity to mobilise on day 1 or sooner and fewer mobilised when compared to the cognitively healthy group (Table 2). Bone protection medication increased for both groups on discharge and in patients from private residences and RACF, but more so in patients from private residences (Table 2).

**Table 2:** Clinical care provided to people with and without cognitive impairment: ≥50 years with surgical repair of a minimal trauma hip fracture who were admitted to a hospital participating in the Australian and New Zealand Hip Fracture Registry (n = 49,063).

Characteristic, n (%)	Cognitively healthy	Impaired cognition or known dementia	*p*-value
	n = 30,027	n = 19,036	
**Pain assessment ≤30 min ED presentation**			<0.0001
≤30 min	16,558 (67.5)	9,524 (61.2)	
>30 min	5,353 (21.8)	3,500 (22.5)	
Not documented	2,615 (10.7)	2,527 (16.3)	
**Analgesia ≤30 min ED presentation**			<0.0001
≤30 min	5,896 (24.3)	3,593 (23.5)	
>30 min	4,852 (20.0)	4,033 (26.4)	
Not required, already given by paramedics	12,430 (51.3)	6,934 (45.4)	
Not required, no pain on assessment	1,058 (4.4)	716 (4.7)	
**Analgesia – nerve block**			<0.0001
Nerve block before theatre	12,076 (41.8)	7,239 (39.5)	
Nerve block in theatre	4,158 (14.4)	3,095 (16.9)	
Both	9,496 (32.9)	6,050 (33.0)	
Neither	3,130 (10.9)	1,933 (10.6)	
**Pre-operative cognitive assessment**			<0.0001
Not assessed	11,605 (45.3)	6,766 (41.5)	
Assessed	14,004 (54.7)	9,539 (58.5)	
**Pre-operative medical assessment**			<0.0001
No assessment	8,295 (28.1)	4,491 (24.0)	
Geriatrician/geriatric team	15,894 (53.9)	11,109 (59.2)	
Physician/physician team	4,980 (16.9)	2,971 (15.8)	
GP	69 (0.2)	23 (0.1)	
Specialist nurse	279 (1.0)	158 (0.8)	
**Time to surgery <48h**^a^	23,278 (80.0)	14,656 (79.5)	0.203
**Anaesthesia**			<0.0001
General	16,504 (55.5)	11,026 (58.4)	
Spinal/regional	8,783 (29.5)	4,505 (23.9)	
General and spinal/regional	4,431 (14.9)	3,311 (17.5)	
Other	44 (0.2)	30 (0.2)	
**Surgical approach**			<0.0001
Fixation (e.g. screws/nails)	17,056 (57.2)	10,654 (56.3)	
Hemiarthroplasty	8,365 (28.0)	7,791 (41.2)	
THR	4,262 (14.3)	350 (1.9)	
Other	161 (0.5)	117 (0.6)	
**Consultant present for surgery**	19,022 (64.2)	11,180 (59.6)	<0.0001
**Restricted weightbearing status**	1,536 (5.2)	559 (3.0)	<0.0001
**For patients not immobile on admission:**			
Given the opportunity to mobilise day-1	27,258 (93.2)	15,720 (89.1)	<0.0001
Mobilised day-1	4,058 (58.4)	1,421 (34.2)	<0.0001
**Bone protection medication on discharge**			<0.0001
No	6,816 (23.6)	3,711 (20.3)	
Calcium and/or vit D	14,290 (49.5)	10,186 (55.6)	
Yes, prescription	7,787 (27.0)	4,432 (24.2)	
**Bone protection medication on discharge in patient from private residences**			<0.0001
No	6,282 (23.9)	1,391 (19.6)	
Calcium and/or vit D	12,884 (49.0)	3,769 (53.2)	
Yes, prescription	7,156 (27.2)	1,921 (27.1)	
**Bone protection medication on discharge in patient from RACF**			<0.0001
No	478 (20.3)	2,282 (20.6)	
Calcium and/or vit D	1,291 (54.9)	6,298 (57.0)	
Yes, prescription	581 (24.7)	2,474 (22.4)	
**Received rehabilitation from acute care**^b^	18,648 (64.1)	6,345 (35.1)	<0.0001
From private residence	17,618 (66.3)	4,532 (64.8)	0.0210
From RACF	897 (38.7)	1,730 (15.9)	<0.0001

*Note.* ED = emergency department, GP = general practitioner, RACF = residential aged care facility.

Missing data or ‘not known’: pain assessment n = 3,508 of 43,785 (variable introduced 2017); analgesia ED n = 4,273 of 43,785 (variable introduced 2017); analgesia nerve block n = 1,886; pre-operative cognitive assessment n = 1,871 of 43,785 (variable introduced 2017); pre-operative medical assessment n = 794; time to surgery n = 1,405; anaesthesia n = 429; type of surgery n = 307; consultant present n = 685; weightbearing n = 356; offered first day walking n = 761; mobilised day-1 n = 1,581 of 12,676 (this variable was introduced in 2020 and is only reported in those mobile preadmission); bone protection medication on discharge n = 1,841 (n = 1,346 from private residences; n = 455 from RACF); received rehabilitation n = 585.

a This has been calculated using date and time variables. The time from first presentation of hip fracture to surgery e.g. in-hospital fracture date, from the first hospital admission date and time if the patient was transferred. n = 132 excluded from data with time to surgery ≥291 h (3SD above mean) and 1,405 were missing due to missing or implausible date/time data.

b Excluding people who had death coded as their discharge destination from acute care.

Significantly fewer people in the cognitively impaired group were transferred to a rehabilitation service although for patients from private residences, the proportion transferred to rehabilitation from the acute ward was similar – 65% with and 66% without cognitive impairment. In patients from RACF, those with cognitive impairment were significantly less likely to transfer to rehabilitation (Table 2).

### 3.3 Outcomes

#### 3.3.1 Delirium, pressure injury and length of stay

Patients in the cognitively impaired group were more likely to have postoperative delirium and develop a new pressure injury than the cognitively healthy group (Table 3). The acute ward length of stay was longer but overall hospital length of stay was shorter in the cognitively impaired group. However, in patients from RACFs, length of stay was shorter for both the acute ward and hospital stay in the cognitively impaired group (Table 3).

**Table 3:** Outcomes for people with and without cognitive impairment: ≥50 years with surgical repair of a low trauma hip fracture who were admitted to a hospital participating in the Australian and New Zealand Hip Fracture Registry (n = 49,063).

Characteristic, n (%), mean ± SD or median [IQR]	Cognitively healthy	Impaired cognition or known dementia	*p*-value
	n = 30,027	n = 19,036	
**Delirium assessment in the week after surgery**			<0.0001
Not assessed	7,693 (37.3)	3,922 (31.2)	
Assessed and not identified	10,149 (49.2)	3,247 (25.0)	
Assessed and identified	2,791 (13.5)	5,839 (44.9)	
**New pressure injury**	832 (2.9)	751 (4.1)	<0.0001
**Length of stay, days**			
Acute ward	8.7 ± 7.8	8.9 ± 7.1	0.0020
	7 [5, 10]	7 [5, 10]	0.0008
Hospital	22.8 ± 19.9	17.9 ± 18.8	<0.0001
	18 [8, 31]	10 [6, 24]	<0.0001
**Length of stay for patients admitted from private residences, days**^a^			
Acute ward	8.7 ± 7.9	10.8 ± 9.0	<0.0001
	7 [5, 10]	8 [5, 13]	<0.0001
Hospital	23.4 ± 20.2	29.4 ± 22.8	<0.0001
	19 [8, 31]	25 [13, 38]	<0.0001
**Length of stay for patients admitted from RACF, days**^b^			
Acute ward	8.6 ± 5.5	7.7 ± 5.2	<0.0001
	7 [5, 10]	6 [5, 9]	<0.0001
Hospital	16.6 ± 14.8	10.9 ± 10.9	<0.0001
	11 [7, 22]	7 [5, 12]	<0.0001
**New transfer to a residential care facility**^c^	2,252 (10.8)	2,467 (46.4)	<0.0001
**30-day mortality**^d^, ^e^	975 (3.4)	2,136 (11.9)	<0.0001
From private residence	737 (2.8)	499 (7.2)	<0.0001
From RACF	226 (9.7)	1,624 (14.9)	<0.0001
**365-day mortality**^d^, ^e^, ^f^	3,683 (15.1)	5,952 (39.2)	<0.0001
From private residence	2,930 (13.2)	1,758 (29.5)	<0.0001
From RACF	725 (36.4)	4,182 (46.7)	<0.0001

*Note.* ASA = The American Society of Anesthesiologists; IQR = interquartile range; THR = total hip replacement; RACF = residential aged care facility.

Missing or not known data: delirium assessment n = 1,887 of 35,528 (variable introduced 2018); pressure injury n = 1,947; length of stay acute ward n = 650 (excluded an additional 61 with LOS ≤ 0 or >364 days); length of stay hospital n = 3,285 (excluded an additional 45 with LOS ≤ 0 or >364 days).

a n = 34,224 for acute and n = 31,898 for hospital length of stay.

b n = 13,692 for acute and n = 13,471 for hospital length of stay.

c people from private residences on admission (n = 34,749) and excluding people who had death or other coded as discharge destination from hospital, n = 3,476 with missing or unknown discharge destination.

d n = 13 cognitively healthy and n = 8 cognitively impaired with implausible deaths, therefore these patients were excluded from mortality analyses.

e South Australian people (n = 2,118) were excluded from mortality analysis as identifiers were not collected for a period which meant they were unable to be linked to the NDI – this is in line with the ANZHFR annual report.

f patients admitted on or after 01 June 2020 were excluded as death data up to June 2021 (Excluded: n = 7,364; n = 4,517 (15.6%) cognitively healthy and n = 2,847 (15.8%) cognitively impaired).

#### 3.3.2 Discharge destination

The discharge destination (from the acute ward and hospital) for patients with and without cognitive impairment is presented in Table 4. Overall, patients in the cognitively impaired group were less likely to be discharged from the acute ward to a private residence and were more likely to be discharged to an RACF or die (Table 3). Patients with cognitive impairment from private residences were less likely to be discharged from the acute ward and hospital to a private residence and were more likely to be discharged to an RACF or die (Table 4). In contrast, a lower proportion of cognitively healthy patients whose usual residence was an RACF were discharged back to the RACF from the acute ward than patients from RACF with cognitive impairment (Table 4).

**Table 4:** Discharge destination from the acute ward and hospital by preadmission cognitive status and usual residence.

Characteristic, n (%)	Whole sample (n = 49,063)			Usual residence = private residence (n = 34,749)			Usual residence = RACF (n = 13,859)		
	Cognitively healthy	Impaired cognition or known dementia	*p*-value	Cognitively healthy	Impaired cognition or known dementia	*p*-value	Cognitively healthy	Impaired cognition or known dementia	*p*-value
	n = 30,027	n = 19,036		n = 27,349	n = 7,400		n = 2,430	n = 11,429	
**Discharge destination acute ward**^a^	n = 29,683	n = 18,795		n = 27,041	n = 7,281		n = 2,408	n = 11,313	
Private residence	5,951 (20.1)	600 (3.2)	<0.0001	5,868 (21.7)	482 (6.6)	<0.0001	49 (2.0)	106 (0.9)	<0.0001
RACF	1,554 (5.2)	9,253 (49.2)		377 (1.4)	849 (11.7)		1,165 (48.4)	8,353 (73.8)	
Rehabilitation - public	15,410 (51.9)	5,647 (30.1)		14,534 (53.8)	4,019 (55.2)		760 (31.6)	1,549 (13.7)	
Rehabilitation - private	3,238 (10.9)	698 (3.7)		3,084 (11.4)	513 (7.1)		137 (5.8)	181 (1.6)	
Other hospital/ward	2,702 (9.1)	1,586 (8.4)		2,488 (9.2)	1,004 (13.8)		177 (7.4)	552 (4.9)	
Deceased	570 (1.9)	731 (3.9)		470 (1.7)	291 (4.0)		91 (3.8)	436 (3.9)	
Short term care RACF (NZ only)	62 (0.2)	54 (0.3)		55 (0.2)	39 (0.5)		<1%^b^	<1%^b^	
Other	196 (0.7)	226 (1.2)		165 (0.6)	84 (1.2)		≈1%^b^	≈1%^b^	
**Discharge destination from hospital**^a^	n = 27,115	n = 17,954		n = 24,638	n = 6,635		n = 2,299	n = 11,148	
Private residence	18,941 (69.9)	3,204 (17.9)	<0.0001	18,638 (75.7)	2,849 (42.9)	<0.0001	204 (8.9)	310 (2.8)	<0.0001
RACF	4,127 (15.2)	12,578 (70.1)		2,252 (9.1)	2,467 (37.2)		1,846 (80.3)	10,025 (89.9)	
Deceased	1,049 (3.9)	1,136 (6.3)		906 (3.7)	541 (8.2)		129 (5.6)	586 (5.3)	
Other	2,998 (11.1)	1,036 (5.8)		2,842 (11.5)	778 (11.7)		120 (5.2)	227 (2.0)	
**Discharge destination from hospital for patients who received rehab**^c^	n = 16,422	n = 5,669	<0.0001	n = 15,528	n = 4,012	<0.0001	n = 800	n = 1,590	<0.0001
Private residence	11,742 (71.5)	2,167 (38.2)		11,557 (74.4)	2,029 (50.6)		126 (15.8)	110 (6.9)	
RACF	2,253 (13.7)	2,697 (47.6)		1,656 (10.7)	1,344 (33.5)		582 (72.8)	1,329 (83.6)	
Deceased	315 (1.9)	204 (3.6)		292 (1.9)	142 (3.5)		21 (2.6)	58 (3.7)	
Other	2,112 (12.9)	601 (10.6)		2,023 (13.0)	497 (12.4)		71 (8.9)	93 (5.9)	
**Discharge destination from hospital for patients who did not receive rehab**^d^	n = 10,089	n = 11,517	<0.0001	n = 8,610	n = 2,319		n = 1,404	n = 9,098	<0.0001
Private residence	7,180 (71.2)	1,029 (8.9)		7,062 (82.0)	813 (35.1)		78 (5.6)	199 (2.2)	
RACF	1,869 (18.5)	9,861 (85.6)		594 (6.9)	1,120 (48.3)		1,261 (89.8)	8,679 (95.4)	
Deceased	161 (1.6)	197 (1.7)		142 (1.7)	107 (4.6)		16 (1.1)	89 (1.0)	
Other	879 (8.7)	430 (3.7)		812 (9.4)	279 (12.0)		49 (3.5)	131 (1.4)	

Note. NZ = New Zealand; RACF = residential aged care facility.

a n = 585 acute ward, n = 3,994 hospital missing or not known discharge destination data for whole sample; n = 427 acute ward, n = 3,476 missing or not known hospital discharge destination data for patients from private residences; n = 138 acute ward, n = 412 missing or not known hospital discharge destination data for patients from RACF.

b suppressed due to small numbers.

c n = 2,902 missing or not known hospital or not known discharge destination data for whole sample; n = 2,610 missing or not known hospital discharge destination data for patients from private residences; n = 237 missing or not known hospital discharge destination data for patients from RACF.

d n = 578 missing or not known hospital discharge destination data for whole sample; n = 482 missing or not known hospital discharge destination data for patients from private residences; n = 65 missing or not known hospital discharge destination data for patients from RACF. Patients who died during their acute stay were excluded from these analyses.

#### 3.3.3 Mortality

In the whole sample, 30- and 365-day mortality were 6.6% (n = 3,111) and 24.4% (n = 9,635), respectively. Mortality (30- and 365-day) by cognitive group and preadmission residential status is presented in Table 3. Thirty- and 365-day mortality were significantly higher in the cognitively impaired group when compared to the cognitively healthy group in the whole sample, in patients from private residences and in patients from RACFs (Table 3). Mortality stratified by age is presented in Supplementary Table 1. When stratified by age group, the cognitively impaired group had significantly higher 30-day mortality in each age group regardless of usual place of residence (Table S1). When stratified by age group, the cognitively impaired group had significantly higher 365-day mortality in each age group in the whole sample and patients from private residences (Table S1). There were mixed findings for 365-day mortality in residents of care facilities by age group (Table S1).

### 3.4 Multivariable associations between cognitive impairment/known dementia and; 1) being malnourished, 2) delirium in the week after surgery, 3) day-1 walking, 4) discharge to an RACF (vs home) in people from private residences and 5) 30-day mortality

**Fig. 2:**
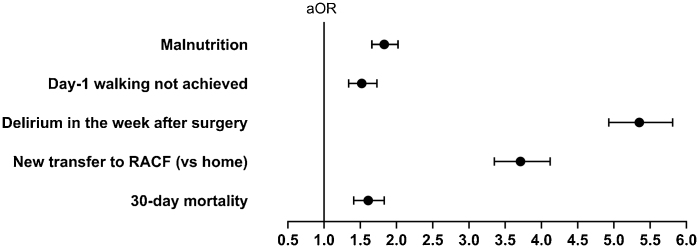
Adjusted odds ratios demonstrating the association between cognitive impairment/known dementia and being malnourished, not achieving day-1 walking (in those who were mobile preadmission), having delirium in the week after surgery (in those who were assessed), having a new transfer to a residential aged care facility (RACF; vs home in people from private residences) and 30-day mortality. ***Note.*** Mixed effects logistic regression models with facility as the random effect. For the outcome malnourished, the model was adjusted for age, sex, usual residence, preadmission mobility and ASA classification (<3 vs ≥3). All other models were adjusted for age, sex, preadmission mobility, The American Society of Anesthesiologists classification (<3 vs ≥3), fracture type and approach to surgical fixation. All models were adjusted for delirium in the week after surgery, except for when delirium was the outcome. All models were adjusted for usual residence, except for when new transfer to an RACF was the outcomes (as this was only for people admitted from private residences). New transfer to an RACF was additionally adjusted for receiving inpatient rehabilitation. Full model details for day-1 walking, delirium, discharge to an RACF and 30-day mortality can be found in Supplementary Table 2.

While adjusting for age, sex, usual residence, preadmission mobility and ASA classification (<3 vs ≥3), the cognitively impaired group had an increased odds of being malnourished (aOR 1.83, 95%CI 1.66, 2.02; Fig. 2).

While adjusting for covariates, the cognitively impaired group had a significantly increased odds of not achieving day-1 walking, having delirium in the week after surgery, having a new transfer to an RACF (in people from private residences) and dying within 30-days (Fig. 2 and Supplementary Table 2).

**Fig. 3:**
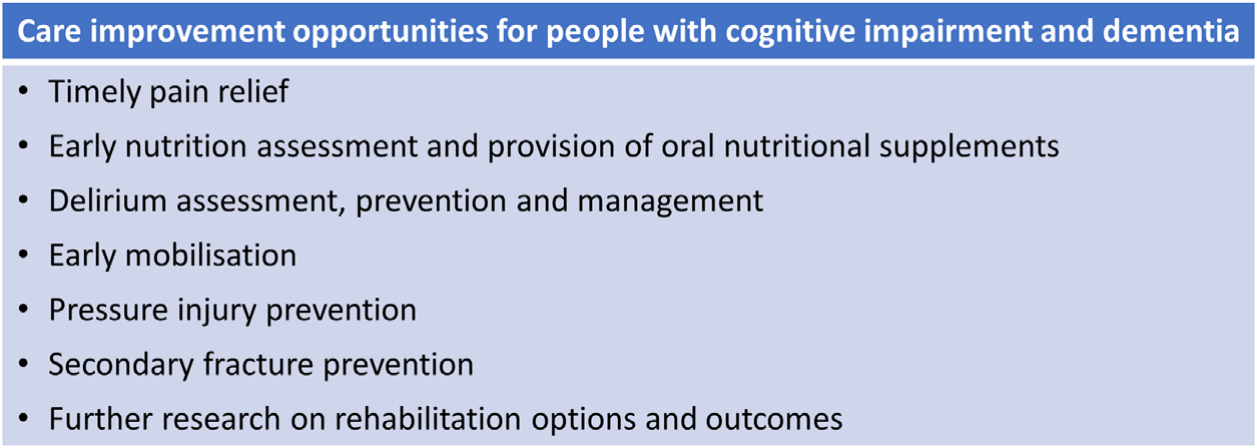
Summary of the study findings: opportunities for care improvements for people with cognitive impairment/dementia. (Please note. Fig. 3 is not exhaustive and focuses on the findings from this study).

Fig. 3 provides a summary of the identified opportunities for care improvements for people with cognitive impairment/dementia who sustain a hip fracture.

## 4 Discussion

This study has identified several important differences in the processes of care and outcomes following hip fracture based on cognitive status. A number of these areas identified are potentially modifiable (e.g. pain management, early mobilisation; Fig. 3) whilst others require more exploration to determine if a different approach to care can alter outcomes (rehabilitation for those who live in an RACF). Of note, in multivariable models adjusting for covariates and facility, cognitive impairment was independently associated with an increased odds of being malnourished, not walking the day after surgery, having a delirium in the week after surgery, having a new transfer to an RACF and dying within 30-days of presentation for a hip fracture.

The observed delay in pain assessment and management in this study is something that needs to be addressed. Effective management of pain is essential from a patient perspective and negatively impacts the hip fracture journey if not adequately managed. For example, pain is a modifiable risk factor for delirium [14]. Assessment of pain can be challenging for health professionals caring for people with cognitive impairment or dementia and there is concern around the use of opiate containing agents and the risk of delirium. Pain in patients with dementia is often under recognised and undertreated in part due to an inability to adequately and/or accurately vocalise symptoms, and the requirement for staff to look for non-verbal signs of pain that may go unnoticed [15]. Use of pain scales designed specifically for people with dementia may be of value (PAINAD or Abbey Pain Scale) and consideration should be given to increasing the pre-hospital use of nerve blocks in hip fracture patients, particularly those in whom there is concern around delirium with the use of systemic opiate containing agents.

Early mobilisation represents another opportunity to improve care, particularly in patients with cognitive impairment considering the independent association with not achieving day-1 walking. Researchers in the UK have demonstrated poorer outcomes (e.g. poorer recovery of the ability to walk, lower odds of discharge and reduced survival at 30-days) in patients who were not mobilised early postoperatively (day of or day after surgery) [9,16]. In the Australian and New Zealand context, while approximately 90% of patients were given the opportunity to mobilise, far fewer actually mobilised on the day of or day after surgery. In a recent Sprint audit, involving 29 hospitals in Australia and 7 in New Zealand from the ANZHFR, delirium/agitation was cited as the most common reason for not mobilising the day of or day after surgery [17]. The proportion of people mobilised early after surgery was significantly lower in patients with cognitive impairment (34%) when compared to cognitively healthy patients (58%) and cognitive impairment was associated with a 52% increased odds of not achieving day-1 walking after adjustment for covariates. This contrasts with data from England and Wales (2014–2016), where 72% of patients with and 81% of patients without dementia were mobilised early [16]. In the UK, the best practice tariff (funding payable if all best practice tariff criteria are met) includes ‘assessed by a physiotherapist the day of or day following surgery’, which raises the importance of mobility and encourages hospitals to provide weekend physiotherapy services. In Australia and New Zealand between 78% and 80% of hospitals report access to weekend therapy services, but how these services operate (e.g. one day, both days) is currently not known [12]. However, the sprint audit indicated that 48% and 14% of hip fracture patients routinely received physiotherapy at the weekend in Australia and New Zealand, respectively [17]. In contrast, 48% and 71% indicated that patients were only seen if they were day-1 after surgery in Australia and New Zealand, respectively [17]. More intensive postoperative physiotherapy including 7-day physiotherapy services have been demonstrated to improve outcomes (function and length of stay) for patients with hip fracture and these samples included people with cognitive impairment [18,19].

Malnutrition was more common in the cognitively impaired group (41% vs 23% of those assessed) and cognitive impairment was independently associated with malnutrition while adjusting for patient characteristics. Whilst this is likely a reflection of the cohorts’ nutrition status at admission, we also know that older people are at risk of developing malnutrition in hospital. Malnutrition is associated with increased mortality and poorer functional outcomes in patients after hip fracture surgery [20]. Identifying and treating malnutrition should be an integral component of gold standard multidisciplinary care, particularly for patients with cognitive impairment and dementia. Nutritional assessment is included in the UK best practice tariff and the Scottish Standards of Care for Hip Fracture Patients but was not a quality indicator in the Australian and New Zealand Hip Fracture Care Clinical Care Standard [10]. However, in the new Hip Fracture Clinical Care Standard, released September 2023, quality indicator 3c will assess the proportion of patients receiving nutritional supplements during their admission for a hip fracture [21].

Delirium predicts poorer outcomes in hospitalised older people [22], a finding which has been further supported by our multivariable models. In this study more than one third of patients were not objectively assessed for the presence or absence of delirium in the week after surgery. In Scotland, delirium screening within 24 h of admission to the ward was 79% in 2019 and in 2022 in the UK, 70% of patients were assessed and not delirious suggesting that Australia and New Zealand have room for improvement in assessing, preventing and managing delirium [23,24]. As demonstrated in this study, those most at risk of delirium are people with underlying cognitive impairment and if risk of delirium is identified it should be followed up with intervention strategies both to prevent and manage delirium. Multicomponent intervention strategies implemented within 24 h of admission for hip fracture have been demonstrated to reduce the incidence of delirium and some studies have demonstrated improved outcomes [25]. In patients with cognitive impairment, two studies graded moderate quality involving multicomponent interventions, reduced delirium incidence in patients with hip fracture and improved outcomes e.g. urinary tract infections, falls and walking ability at 4-months [[26], [27], [28]]. Delirium prevention strategies should be routinely implemented for all patients with hip fracture.

The variation in care demonstrated in the current study provides evidence to support an intervention aimed at ameliorating these differences, with the hope of improving the demonstrated increase in hospital acquired complications (e.g. increased postoperative delirium and pressure injury) and poorer outcomes (e.g. higher mortality and new transfer to residential care) for this group. The increase in hospital acquired complications also highlights some potential avenues for interventions. While we can improve the care and environment we provide for people with cognitive impairment in hospital, it is unlikely that this will significantly impact their cognitive impairment. However, delirium is potentially modifiable and previous research suggests that simple strategies can help prevent delirium [29].

One of the most striking features of this study is the impact of cognition on new transfer to a RACF. More than one third of patients from private residences with cognitive impairment were discharged from hospital to an RACF, and when excluding patients who had died this increased to >40%. In adjusted analyses, the cognitively impaired group had a 3.7 times increased odds of new transfer to an RACF than patients without cognitive impairment. Whilst the proportion of people from the community with and without cognitive impairment accessing rehabilitation was similar (65% vs 64%), those with cognitive impairment from the community who accessed rehabilitation had a 36% reduced odds of being discharged to an RACF in adjusted analysis. Also of note in this study is the small percentage of patients from RACFs that access rehabilitation. It is not possible to say whether this reflects the limited reserves of the individuals to make meaningful gains in function or evidence of inequitable access to services from which a person might benefit. Interestingly, in the recent ANZHFR Sprint audit which asked hospitals about criteria for acceptance to a rehabilitation service, impaired cognition was not a common reason for declining access [17]. The most common reason cited was lack of rehabilitation goals [17].

The strengths of this manuscript include the large sample size which includes binational clinical data linked to administrative death data. This increases the generalisability of our results to Australia and New Zealand broadly. However, this means that it is not possible to know if the findings are generalisable to health systems internationally. There are also some further limitations to consider. The study is retrospective and the data is often being entered by clinical staff who are time poor and often without specific funding for their ANZHFR data entry role. This may increase the risk of data entry errors and means that researchers are not able to validate the data against accessible records [30]. However, data entry validation rules and guidance have evolved over time to help reduce the occurrence of errors. New variables have been introduced over the years, which means that for some variables, we have a reduced sample size. Finally, in our multivariable models there may be factors that influence the dependent variables that are not accounted.

We have identified several aspects of care that could be improved upon for patients with cognitive impairment – pain assessment and management, early mobility, delirium assessment, prevention and management, nutrition assessment and management, and prescription of bone protection medication. Consistent with previous literature we have also demonstrated increased hospital acquired complications (e.g. delirium and pressure injuries) and poorer outcomes (e.g. mortality and new transfer to RACF) in people with cognitive impairment. We have also identified questions that require further exploration and particularly around the issue of access to rehabilitation after hip fracture in those who are cognitively impaired including those from RACFs. Improving the multidisciplinary care provided to people with dementia after hip fracture, ensuring it is tailored to the needs of the person with dementia, will hopefully reduce hospital complications and improve outcomes.

## Conflicts of interest

JCTC is Co-Chair and a member of the Steering Group of the ANZHFR (2015-current). IH was Co-Chair the ANZHFR (2016–2021). MET is a part-time data analyst for the ANZHFR (2023) and as part of this role a member of the Research and Data Management Subcommittees, however this manuscript was not written as part of this role. LH is on the Research Subcommittee of the ANZHFR.

## Funding information

MET receives partial salary funding from Centre for Research Excellence in the Prevention of Fall-related Injuries (NHMRC).

## Data availability statement

Data may be obtained from a third party (the Australian and New Zealand Hip Fracture Registry) and are not publicly available. Data access requests need to be submitted to clinical@anzhfr.org and approval from an ethical review board is required.

## Footnotes

Appendix A Supplementary material related to this article can be found, in the online version, at doi:https://doi.org/10.1016/j.jnha.2023.100030.

## References

[1] Australian Institute of Health and Welfare. Hip fracture incidence and hospitalisations in Australia 2015-2016 2018 [Available from: https://www.aihw.gov.au/reports/injury/hip-fracture-incidence-in-australia-2015-16/summary.

[2] Hou M., Zhang Y., Chen A.C., Liu T., Yang H., Zhu X. (2021). The effects of dementia on the prognosis and mortality of hip fracture surgery: a systematic review and meta-analysis. Aging Clin Exp Res.

[3] Harvey L., Mitchell R., Brodaty H., Draper B., Close J. (2016). Differing trends in fall-related fracture and non-fracture injuries in older people with and without dementia. Arch Gerontol Geriatr.

[4] Mitchell R., Draper B., Brodaty H., Close J., Ting H.P., Lystad R. (2020). An 11-year review of hip fracture hospitalisations, health outcomes, and predictors of access to in-hospital rehabilitation for adults >= 65 years living with and without dementia: a population-based cohort study. Osteoporos Int.

[5] Bail K., Berry H., Grealish L., Draper B., Karmel R., Gibson D. (2013). Potentially preventable complications of urinary tract infections, pressure areas, pneumonia, and delirium in hospitalised dementia patients: retrospective cohort study. BMJ Open.

[6] Bail K., Goss J., Draper B., Berry H., Karmel R., Gibson D. (2015). The cost of hospital-acquired complications for older people with and without dementia; a retrospective cohort study. BMC Health Serv Res.

[7] Independent Hospital Pricing Authority, Authority IHP (2021). Pricing framework for Australian Public Hospital Services 2022–23.

[8] Mosk C.A., Mus M., Vroemen J.P., van der Ploeg T., Vos D.I., Elmans L.H. (2017). Dementia and delirium, the outcomes in elderly hip fracture patients. Clin Interv Aging.

[9] Sheehan K.J., Goubar A., Martin F.C., Potter C., Jones G.D., Sackley C. (2021). Discharge after hip fracture surgery in relation to mobilisation timing by patient characteristics: linked secondary analysis of the UK National Hip Fracture Database. BMC Geriatr.

[10] Australian Commission on Safety and Quality in Health Care (2016).

[11] The Australian and New Zealand Hip Fracture Registry. ANZHFR Annual Report 2021. 2021.

[12] Australian and New Zealand Hip Fracture Registry. ANZHFR Annual Report 2022. 2022.

[13] Smith T.O., Gilbert A.W., Sreekanta A., Sahota O., Griffin X.L., Cross J.L. (2020). Enhanced rehabilitation and care models for adults with dementia following hip fracture surgery. Cochrane Database Syst Rev.

[14] Sampson E.L., West E., Fischer T. (2020). Pain and delirium: mechanisms, assessment, and management. Eur Geriatr Med.

[15] International Association in the Study of Pain. Pain Assessment in Dementia: International Association in the Study of Pain; 2021 [Fact sheet]. Available from: https://www.iasp-pain.org/resources/fact-sheets/pain-assessment-in-dementia/.

[16] Goubar A., Martin F.C., Potter C., Jones G.D., Sackley C., Ayis S. (2021). The 30-day survival and recovery after hip fracture by timing of mobilization and dementia: a UK database study. Bone Joint J.

[17] Australian and New Zealand Hip Fracture Registry. Acute Rehabilitation Sprint Audit Sydney: Australian and New Zealand Hip Fracture Registry; 2022 [Available from: https://anzhfr.org/sprintaudits/.

[18] Kimmel L.A., Liew S.M., Sayer J.M., Holland A.E. (2016). Hip4Hips (High Intensity Physiotherapy for hip fractures in the acute hospital setting): a randomised controlled trial. Med J Aust.

[19] Goubar A., Ayis S., Beaupre L., Cameron I.D., Milton-Cole R., Gregson C.L. (2022). The impact of the frequency, duration and type of physiotherapy on discharge after hip fracture surgery: a secondary analysis of UK national linked audit data. Osteoporos Int.

[20] Foo M.X.E., Wong G.J.Y., Lew C.C.H. (2021). A systematic review of the malnutrition prevalence in hospitalized hip fracture patients and its associated outcomes. J Parenter Enteral.

[21] Australian Commission on Safety and Quality in Health Care (2023).

[22] Welch C., McCluskey L., Wilson D., Chapman G.E., Jackson T.A., Treml J. (2019). Delirium is prevalent in older hospital inpatients and associated with adverse outcomes: results of a prospective multi-centre study on World Delirium Awareness Day. BMC Med.

[23] Public Health Scotland (2020).

[24] National Falls and Fragility Fracture Audit Programme. National Hip Fracture Database: Assessment benchmark summary 2022: National Falls and Fragility Fracture Audit Programme; 2022 [updated 22/02/2023. Available from: https://www.nhfd.co.uk/20/nhfdcharts.nsf/fmBenchmarks?readform.

[25] Oberai T., Laver K., Crotty M., Killington M., Jaarsma R. (2018). Effectiveness of multicomponent interventions on incidence of delirium in hospitalized older patients with hip fracture: a systematic review. Int Psychogeriatr.

[26] Stenvall M., Berggren M., Lundstrom M., Gustafson Y., Olofsson B. (2012). A multidisciplinary intervention program improved the outcome after hip fracture for people with dementia–subgroup analyses of a randomized controlled trial. Arch Gerontol Geriatr.

[27] Schnitker L., Nović A., Arendts G., Carpenter C.R., LoGiudice D., Caplan G.A. (2020). Prevention of delirium in older adults with dementia: a systematic literature review. J Gerontol Nurs.

[28] Freter S., Koller K., Dunbar M., MacKnight C., Rockwood K. (2017). Translating delirium prevention strategies for elderly adults with hip fracture into routine clinical care: a pragmatic clinical trial. J Am Geriatr Soc.

[29] Mudge A.M., McRae P., Banks M., Blackberry I., Barrimore S., Endacott J. (2022). Effect of a ward-based program on hospital-associated complications and length of stay for older inpatients: the cluster randomized CHERISH trial. JAMA Internal Med.

[30] Tan A.C., Armstrong E., Close J., Harris I.A. (2019). Data quality audit of a clinical quality Registry: a generic framework and case study of the Australian and New Zealand Hip Fracture Registry. BMJ Open Qual.

