# Supplementary material for: Variation in care and outcomes for people after hip fracture with and without cognitive impairment; results from the Australian and New Zealand Hip Fracture Registry
Source: J Nutr Health Aging. 2024 Jan 4;28(2):100030. doi: 10.1016/j.jnha.2023.100030 (PMC12877269; doi:10.1016/j.jnha.2023.100030)
Supplement: Supplementary file 1 [file mmc1.docx]

**Supplementary Table 1.** Thirty- and 365-day mortality by preadmission cognitive status, usual residence and age group in patients who had surgery for a low trauma hip fracture (n=49,063)

|  | Whole sample | | |  | Usual residence = private residence | | |  | Usual residence = RACF | | |
| --- | --- | --- | --- | --- | --- | --- | --- | --- | --- | --- | --- |
|  | Cognitively healthy (n=28,897)^a,b^ | Cognitively impaired (n=18,027)^a,b^ | *p*-value^c^ |  | Cognitively healthy (n=26,327)^b,d^ | Cognitively impaired (n=6,962)^b,d^ | *p*-value^c^ |  | Cognitively healthy (n=2,427)^b,e^ | Cognitively impaired (n=11,424)^b,e^ | *p*-value^c^ |
| 30-day mortality (all ages) | 975 (3.4) | 2,136 (11.9) | <0.0001 |  | 737 (2.8) | 499 (7.2) | <0.0001 |  | 226 (9.7) | 1,624 (14.9) | <0.0001 |
| 30-day mortality by age group | | |  |  |  |  |  |  |  |  |  |
| 50-64 years | 12 (0.5) | 11 (3.2) | <0.0001 |  | 12 (0.5) | 8 (3.8) | 0.0001 |  | 6 (3.2)^f^ | 51 (7.6)^f^ | 0.0309^b^ |
| 65-74 years | 97 (1.7) | 79 (6.5) | <0.0001 |  | 90 (1.7) | 30 (4.9) | <0.0001 |  |  |  |  |
| 75-84 years | 258 (2.7) | 468 (9.2) | <0.0001 |  | 220 (2.4) | 122 (5.5) | <0.0001 |  | 36 (7.3) | 342 (12.2) | 0.0018 |
| 85-94 years | 475 (5.0) | 1,200 (12.7) | <0.0001 |  | 343 (4.2) | 271 (8.0) | <0.0001 |  | 128 (10.2) | 925 (15.5) | <0.0001 |
| 95+ years | 133 (8.6) | 378 (19.3) | <0.0001 |  | 72 (6.3) | 68 (12.9) | <0.0001 |  | 56 (14.3) | 306 (21.6) | 0.0014 |
| 365-day mortality (all ages)^g^ | 3,683 (15.1) | 5,952 (39.2) | <0.0001 |  | 2,930 (13.2) | 1,758 (29.5) | <0.0001 |  | 725 (36.4) | 4,182 (46.7) | <0.0001 |
| 365-day mortality by age group^g^ | | |  |  |  |  |  |  |  |  |  |
| 50-64 years | 124 (6.0) | 38 (13.5) | <0.0001 |  | 113 (5.6) | 23 (13.5) | <0.0001 |  | 10 (31.3) | 12 (13.8) | 0.0296 |
| 65-74 years | 419 (8.9) | 250 (24.9) | <0.0001 |  | 381 (8.4) | 98 (19.3) | <0.0001 |  | 35 (26.3) | 152 (31.9) | 0.2144 |
| 75-84 years | 1,011 (12.4) | 1,422 (33.3) | <0.0001 |  | 878 (11.5) | 461 (24.6) | <0.0001 |  | 127 (30.1) | 949 (40.3) | <0.0001 |
| 85-94 years | 1,695 (20.8) | 3,360 (42.2) | <0.0001 |  | 1,281 (18.3) | 945 (33.0) | <0.0001 |  | 401 (37.6) | 2,397 (47.5) | <0.0001 |
| 95+ years | 434 (32.8) | 882 (53.1) | <0.0001 |  | 277 (28.4) | 205 (44.9) | <0.0001 |  | 152 (45.0) | 672 (56.2) | 0.0002 |

Note. RACF = residential aged care facility

^a^n=13 cognitively healthy and n=8 cognitively impaired with implausible deaths, therefore these patients were excluded from mortality analyses

^b^South Australian people (n=2,118) were excluded from mortality analysis as identifiers were not collected for a period which meant they were unable to be linked to the NDI – this is in line with the ANZHFR annual report

^c^Chi-square or Fisher’s exact test

^d^n=9 cognitively healthy and n=4 cognitively impaired with implausible deaths, therefore these patients were excluded from mortality analyses

^e^n=3 cognitively healthy and n=4 cognitively impaired with implausible deaths, therefore these patients were excluded from mortality analyses

^f^50-74 years of age

^g^patients admitted on or after 01 June 2020 excluded as death data up to June 2021 (Excluded: n=7,364; n=4,517 (15.6%) cognitively healthy and n=2,847 (15.8%) cognitively impaired)

**Supplementary Table 2.** Multivariable mixed effects logistic regression models with facility as the random effect were used to examine the association between cognitive impairment/known dementia and; not achieving day-1 walking; delirium in the week after surgery; discharge to RACF (vs home) in people from private residences; and 30-day mortality

|  | Did not achieved first day walking in those who were mobile preadmission  (n=11,095)^a^ | |  | Delirium in the week following surgery in those assessed (n=22,026)^b^ | | | |  | New transfer to RACF (vs home)  (n=34,749)^c^ | |  | 30-day mortality in the whole sample  (n=49,063)^d^ | | | |  |  |
| --- | --- | --- | --- | --- | --- | --- | --- | --- | --- | --- | --- | --- | --- | --- | --- | --- | --- |
|  | n=9,620^a^  OR 95% CI | *p*-value |  | n=19,090^b^  OR 95% CI | *p*-value | |  | | n=16,020^c^  OR 95% CI | *p*-value |  | | n=27,733^d^  OR 95% CI | | *p*-value | |  |
| Age, y | 1.01 (1.00, 1.01) | 0.1027 |  | 1.03 (1.03, 1.04) | <0.0001 | |  | | 1.07 (1.06, 1.08) | <0.0001 |  | | 1.05 (1.04, 1.06) | | <0.0001 | |  |
| Female | 0.99 (0.89, 1.09) | 0.7967 |  | 0.77 (0.72, 0.83) | <0.0001 | |  | | 0.86 (0.77, 0.95) | 0.0026 |  | | 0.45 (0.40, 0.50) | | <0.0001 | |  |
| Usual residence (ref = private residence) | |  |  |  |  | |  | | n/a | n/a |  | |  | |  | |  |
| RACF | 1.74 (1.51, 2.00) | <0.0001 |  | 1.25 (1.15, 1.37) | <0.0001 | |  | | n/a | n/a |  | | 1.85 (1.62, 2.10) | | <0.0001 | |  |
| Other | 1.17 (0.52, 2.63) | 0.7100 |  | 0.97 (0.58, 1.63) | 0.9140 | |  | | n/a | n/a |  | | 1.45 (0.61, 3.40) | | 0.3996 | |  |
| Preadmission mobility (ref = unaided) | |  |  |  |  | |  | |  |  |  | |  | |  | |  |
| stick or crutch | 1.30 (1.12, 1.51) | 0.0005 |  | 1.09 (0.97, 1.22) | 0.1467 | |  | | 1.45 (1.27, 1.66) | <0.0001 |  | | 1.10 (0.90, 1.34) | | 0.3417 | |  |
| two aids/frame | 1.63 (1.45, 1.83) | <0.0001 |  | 1.11 (1.02, 1.21) | 0.0128 | |  | | 2.07 (1.86, 2.31) | <0.0001 |  | | 1.57 (1.37, 1.80) | | <0.0001 | |  |
| immobile | n/a | n/a |  | 1.01 (0.83, 1.24) | 0.9216 | |  | | 2.37 (1.68, 3.35) | <0.0001 |  | | 1.78 (1.40, 2.28) | | <0.0001 | |  |
| ASA ≥3 | 1.97 (1.72, 2.26) | <0.0001 |  | 1.71 (1.53, 1.92) | <0.0001 | |  | | 2.15 (1.84, 2.50) | <0.0001 |  | | 2.65 (2.00, 3.51) | | <0.0001 | |  |
| Fracture type (ref = Intracapsular undisplaced/impacted displaced) | | | | |  | |  | |  |  |  | |  | |  | |  |
| Intracapsular displaced | 1.17 (1.01, 1.36) | 0.0377 |  | 1.07 (0.96, 1.19) | 0.2359 | |  | | 1.16 (0.99, 1.35) | 0.0595 |  | | 1.07 (0.90, 1.27) | | 0.4325 | |  |
| Per/intertrochanteric | 1.93 (1.65, 2.25) | <0.0001 |  | 1.28 (1.15, 1.44) | <0.0001 | |  | | 1.30 (1.12, 1.52) | 0.0008 |  | | 1.21 (1.01, 1.45) | | 0.0366 | |  |
| Subtrochanteric | 2.56 (2.03, 3.22) | <0.0001 |  | 1.27 (1.07, 1.51) | 0.0058 | |  | | 1.28 (1.02, 1.61) | 0.0351 |  | | 1.35 (1.05, 1.73) | | 0.0183 | |  |
| Time to surgery ≥48h | 1.26 (1.11, 1.43) | 0.0003 |  | 1.07 (0.98, 1.17) | 0.1450 | |  | | 1.18 (1.05, 1.33) | 0.0053 |  | | 1.25 (1.11, 1.42) | | 0.0004 | |  |
| Surgical fixation (ref = fixation) | |  |  |  |  | |  | |  |  |  | |  | |  | |  |
| Hemiarthroplasty | 1.51 (1.29, 1.77) | <0.0001 |  | 1.39 (1.24, 1.56) | <0.0001 | |  | | 0.98 (0.84, 1.15) | 0.8339 |  | | 1.02 (0.86, 1.21) | | 0.8387 | |  |
| Total hip replacement | 0.98 (0.78, 1.20) | 0.8154 |  | 0.96 (0.81, 1.15) | 0.6730 | |  | | 0.35 (0.26, 0.46) | <0.0001 |  | | 0.42 (0.27, 0.66) | | 0.0001 | |  |
| Other | 2.11 (0.83, 5.40) | 0.1190 |  | 1.14 (0.67, 1.94) | 0.6329 | |  | | 2.50 (1.30, 4.79) | 0.0058 |  | | 0.60 (0.25, 1.44) | | 0.2533 | |  |
| Not offered first day mobilisation | n/a | n/a |  | 1.63 (1.44, 1.86) | <0.0001 | |  | | 1.28 (1.08, 1.53) | 0.0053 |  | | 2.52 (2.19, 2.90) | | <0.0001 | |  |
| Delirium (ref = assessed and not present) | | |  |  |  | |  | |  |  |  | |  | |  | |  |
| Assessed and present | 1.73 (1.52, 1.96) | <0.0001 |  | n/a | n/a | |  | | 1.88 (1.66, 2.12) | <0.0001 |  | | 1.74 (1.52, 2.00) | | <0.0001 | |  |
| Not assessed | 1.27 (1.12, 1.44) | 0.0002 |  | n/a | n/a | |  | | 1.09 (0.96, 1.23) | 0.1980 |  | | 1.58 (1.37, 1.84) | | <0.0001 | |  |
| Cognitive impairment or known dementia | 1.52 (1.34, 1.73) | <0.0001 |  | 5.35 (4.93, 5.81) | <0.0001 | |  | | 3.71 (3.35, 4.12) | <0.0001 |  | | 1.61 (1.41, 1.83) | | <0.0001 | |  |
| Received rehabilitation | n/a | n/a |  | n/a | n/a | |  | | 0.64 (0.58, 0.72) | <0.0001 |  | | n/a | | n/a | |  |
| AUC | 0.80 (0.79, 0.81) | |  | 0.81 (0.80, 0.82) | |  | | | 0.84 (0.83, 0.85) | |  | | | 0.80 (0.79, 0.81) | | | |

*Note.* ASA = The American Society of Anesthesiologists; AUC = area under the curve; RACF = residential aged care facility

^a^n=35,979 were admitted before data collection in 2020; n=1,581 had missing or unknown first day walking status; n=408 were immobile on admission; n=1,475 had missing unknown covariate data

^b^n=13,535 were admitted before data collection in 2018; n=1,887 had missing or unknown delirium outcome; n=11,615 did not have a delirium assessment and were excluded from the model; n=2,936 with missing or unknown covariate data

^c^In people from private residences (n=34,749), n=3,476 with not known or missing discharge residence; n=1,447 were coded as deceased and therefore excluded; n=3,620 coded as other and therefore excluded; n=10,186 with missing or unknown covariate data (some of the missing data is because the data was not collected in earlier years e.g. delirium collected from 2018)

^d^South Australian people (n=2,118) were excluded from mortality analysis as identifiers were not collected for a period which meant they were unable to be linked to the NDI – this is in line with the ANZHFR annual report; n=21 implausible deaths were excluded; n=19,191 with missing or not known covariate data (some of the missing data is because the data was not collected in earlier years e.g. delirium collected from 2018)
